# Effects of Civilization

**Published:** 1856-04

**Authors:** 


### EFFECTS OF CIVILIZATION.

An exchange inquires, "If it is the effect of civilization's vices, that the *Sandwich Islands*, which seventy years ago, contained a population of a third of a million, now number only seventy-two thousand souls?" These effects are not the result of civilization, they are the result of the want of true civilization, of the unbridled depravity of the human heart; a depravity, as inseparable from savage, as from civilized nature. Sin brings crime, and sin and crime bring ruin and death to nations as well as to individuals. National destruction as the result of unrestrained indulgence in the appetites and passions of our nature, is not peculiar to any country or clime, or kindred. Some men have gone still further, and inquired if such an amazing decimation is not attributable to christianizing teachings; and some, with more than human malignity, and among them a woman, have directly charged, that these evils are the natural results of missionary operations. Among these writings, which have already passed to their deserved infamy, are some of *Melville's* senseless and malignant ravings. How wonderful is it, and yet what a sweeping proof of the full truth of the Bible declaration, that *the heart is deceitful above all things, and desperately wicked*, to see educated men using the power which culture, talent and position give them in endeavoring to sap the foundation of our holy religion, whose whole tendency is to elevate, refine, and happify, not only for time, but for eternity, a religion whose teachings are purity itself, and to which these very writers owe it, that they themselves are not as savage in nature, and as beastly in practice, as the worst of the cannibals with whom they have associated.

The true reason of the effects inquired into is, that against the strongest appeals and protests to the contrary, the French government over-rode all municipal law, and under the guns of their men of war, compelled the feeble and peaceful authorities of the Sandwich Islands, to admit their brandies into their towns, to brutify the more ignorant, compelled them to permit bands of reckless sailors to come on shore, and forcibly take aboard their ships, the daughters and the mothers of the helpless Islanders, and in the unrestricted gratification of their lowest natures, not only sunk them to deeper depths, morally, but physically imparted diseases, which in that climate, the remedies available in European countries cannot remove. Hence the ruin, not *because*, but in spite of, christianity's teachings.

